# Community health workers' perspectives on integrating into school settings to support student health

**DOI:** 10.3389/fpubh.2023.1187855

**Published:** 2023-06-21

**Authors:** Nicole Yao, Monica Kowalczyk, LaToya Gregory, Jeannine Cheatham, Tarrah DeClemente, Kenneth Fox, Stacy Ignoffo, Anna Volerman

**Affiliations:** ^1^Department of Medicine, University of Chicago, Chicago, IL, United States; ^2^Department of Pediatrics, University of Chicago, Chicago, IL, United States; ^3^Office of Student Health and Wellness, Chicago Public Schools, Chicago, IL, United States; ^4^Sinai Urban Health Institute, Chicago, IL, United States

**Keywords:** child, community health workers, healthy environments, qualitative analysis, school setting, student health

## Abstract

**Introduction:**

While schools represent key venues for supporting health, they continue to experience gaps in health resources. The integration of community health workers (CHWs) into schools has the potential to supplement these resources but has been underexplored. This study is the first to examine perspectives of experienced CHWs about how CHWs can be applied in school settings to support student health.

**Methods:**

This qualitative study involved conducting semi-structured interviews focused on implementation of CHWs in schools with individuals who held positions aligned with the CHW scope of work. De-identified transcripts were analyzed, and codes were organized into domains and themes.

**Results:**

Among 14 participants, seven domains emerged about the implementation of CHWs in schools: roles and responsibilities, collaborations, steps for integration, characteristics of successful CHWs, training, assessment, and potential challenges. Participants shared various potential responsibilities of school-based CHWs, including educating on health topics, addressing social determinants of health, and supporting chronic disease management. Participants emphasized the importance of CHWs building trusting relationships with the school community and identified internal and external collaborations integral to the success of CHWs. Specifically, participants indicated CHWs and schools should together determine CHWs' responsibilities, familiarize CHWs with the school population, introduce CHWs to the school community, and establish support systems for CHWs. Participants identified key characteristics of school-based CHWs, including having familiarity with the broader community, relevant work experience, essential professional skills, and specific personal qualities. Participants highlighted trainings relevant to school-based CHWs, including CHW core skills and health topics. To assess CHWs' impact, participants proposed utilizing evaluation tools, documenting interactions with students, and observing indicators of success within schools. Participants also identified challenges for school-based CHWs to overcome, including pushback from the school community and difficulties related to the scope of work.

**Discussion:**

This study identified how CHWs can have a valuable role in supporting student health and the findings can help inform models to integrate CHWs to ensure healthy school environments.

## 1. Introduction

Childhood chronic conditions have become more prevalent within the past five decades (1). Youth who identify as non-Hispanic Black or Hispanic or from low socioeconomic backgrounds are at greater risk for developing chronic health conditions and experiencing greater morbidity (2, 3). Along with negative health outcomes, children with chronic illnesses are more likely to have lower educational achievement as compared to those without them (4). Further, childhood chronic conditions are associated with increased morbidity and mortality in adulthood (5). Thus, it is essential for children with chronic illnesses to have access to healthcare, especially for vulnerable groups.

Outside of the healthcare system, schools—where youth spend a majority of their waking hours—are recognized as importance venues to promote a culture of health by providing chronic disease management and preventive services to students (6). Impacts of school-based health services include improvement in academic performance, increased access and utilization of health services, as well as better management of health conditions among students (7, 8). Such services are crucial to health, however, many US schools are unable to provide comprehensive health services, including screening, treatment, and linkage to community-based resources, due to school nurse shortages and unsustainable health programming (9, 10). School nurses play an important role in fostering the wellbeing of the school community, including offering direct care to students, educating students on health, conducting health screenings and referrals, leading school health policies, and managing the system of care within schools (9, 11). However, the majority of school nurses work in multiple schools with a ratio of one nurse for every 950 students, impacting their workload (12). Additionally, 11.4% of public schools have no school nursing (9). Other barriers to sustainable health programming in schools include insufficient resources, such as limited funding as well as a lack of knowledge and confidence among teachers when delivering health education outside their typical expertise (10).

To address the deficiencies in school health services, the integration of community health workers (CHWs) into school settings can support student health and foster a culture of health within school communities (13). Community members and/or individuals who possess a thorough understanding of the community can become CHWs who serve as culturally competent liaisons between health and social services and the community (14), supporting education, counseling, navigation, and advocacy (15). CHW programs have been utilized globally to address health disparities within clinical and community settings (16–19). Within clinical settings, CHWs support clinicians to tailor care through their unique connections with patients to understand their needs and situation (20). Within community settings, CHWs provide health education and connect community members to local health and social services (15). Such CHW interventions have shown positive outcomes, including improving chronic health management among adults (21, 22) and children (21, 23–26), connecting clients to resources (27, 28), and promoting healthy behaviors (17, 29).

Although there is strong evidence for the positive impact of CHWs and the CHW workforce is expanding (30), the implementation of CHW interventions in schools has been underexplored (31). This study is the first to examine the perspectives of experienced CHWs about how CHWs can be integrated and utilized in US school settings to work as part of school-based health teams to support student health and healthy school environments. Although CHWs have been integrated in a few US schools and school-based health centers (32, 33), it remains an uncommon practice globally that requires guidance, particularly from the perspective of seasoned CHWs.

## 2. Methods

### 2.1. Study design

This qualitative study was carried out within an academic-community partnership between an academic medical center, public school district, and community-based research institute in Chicago. Semi-structured interviews were conducted with experienced CHWs regarding the integration and implementation of CHWs in the school setting. It focused on CHWs serving the Chicagoland area as the findings would inform program implementation in Chicago schools. This study was deemed exempt by University of Chicago's Institutional Review Board (IRB21-1786).

### 2.2. Population

Participants were individuals who had work titles or positions that aligned with the CHW's scope of work. Participants were recruited through emails disseminated within CHW-focused organizations and networks in the Chicagoland area. Interested individuals contacted the study team directly and were asked to confirm their work background and/or training to qualify for the study. This study utilized snowball sampling with participants asked at the end of their interview to recommend other potentially eligible CHWs.

### 2.3. Data collection

One-on-one interviews were conducted and audio-recorded via Zoom between January and April 2022. All interviews were conducted by a research project coordinator with a master's degree in public health and four years of qualitative methods experience. The coordinator was employed by the academic medical center and did not have any influence on the CHWs' employment or programs. Verbal consent was obtained prior to the start of interviews.

An interview guide was utilized with questions under six key topics: potential roles and responsibilities of CHWs in schools, qualities and skills needed for CHWs in schools, collaborations within the school community, preparation for integration, methods for assessing the intervention, and challenges CHWs in school may face. Each interview lasted 60–120 min. Participants received $50 e-gift cards for completing the interview. Interviews continued until data saturation was reached. Interviews were transcribed and de-identified by the coordinator to maintain anonymity.

### 2.4. Data analysis

Interview transcripts were analyzed using inductive reasoning with thematic analysis based in grounded theory (34, 35). For the first five interviews, four research team members (AV, LG, MK, NY) independently read and coded one interview at a time and then met to compare codes and resolve discrepancies through discussion. Codes from the first five interviews were organized into themes and domains, with domains adapted from the interview guide's key topics. This framework was applied by three researchers (LG, MK, NY) to the remaining interviews, with themes further refined through iterative discussions. Once the final thematic framework was developed, all transcripts were re-coded by two researchers per interview. Discrepancies were resolved through discussion. Dedoose software (Version 9.0.46) was used for analysis.

## 3. Results

Fourteen individuals participated in the study (Table 1). Titles varied, including CHW, CHW supervisor, communicable disease investigator, and COVID-19 response worker. Participants had a mean of 7.5 years of experience (range = 0.5-22 years). The majority of CHWs worked within health systems (*n* = 11, 78.5%) and served populations in Chicago's west side (*n* = 8, 57.1%) and south side (*n*= 8, 57.1%).

**Table 1:** Characteristics of participating community health workers (CHWs)^*^.

**Characteristics**	***N* (%)**
**Years of experience**	
Mean (SD)	8.0 (6.1)
Median (IQR)	7.5 (9)
Range	0.5 – 22
**Organizational affiliation**	
Health systems	11 (78.5)
Health department	1 (7.1)
Religious institution	1 (7.1)
Other community-based organization	1 (7.1)
**Chicago population served**	
Northwest side	2 (14.3)
South side	8 (57.1)
West side	8 (57.1)

^*^Participants had work titles or positions that aligned with scope of work of community health workers.

Seven domains emerged for incorporating CHWs in schools: roles and responsibilities of CHWs in schools; collaborations to support work; key steps for integration; characteristics of successful CHWs; training for CHWs in schools; assessment of progress or impact; and potential challenges (Table 2).

**Table 2:** Domains, themes, and quotes from experienced community health workers (CHWs) about CHW integration in schools.

**Domain**	**Theme**	**Illustrative quotes**
Roles and responsibilities of community health workers in schools	Provide education • Educate students, families, and staff on chronic conditions; general health (e.g., nutrition, vaccines, primary care); community resources; school health policy (e.g., 504 plans)	“The first one that comes to mind would be sex health. I feel like so much of what kids learn about like sex and health happens at school with their peers because a lot of people aren't talking to their parents. I'm wondering if that [community health workers in school] could be a really good avenue for that because it could be a trusted source and a safe place because it's someone who you know is knowledgeable but is not a parent and not a family member. It's just someone who's there who could answer questions for you, so I think that would definitely be a good fit.” (Interview 11) “When I used to do that, go into the schools, I would go to the [parent teacher association] meetings and talk about my role with the parent and let them know that I would be in the schools on this day and that day. I would be there to assist the children with understanding their medications and how to take it. I didn't like bringing kids out separately who were sick, like who had asthma. You do it as a teachable moment for the whole class. You have those moments. Can I come in and speak to the class? You go and talk to the class, so everybody is aware because not only the person who has asthma should know about how to control it but those who are around him should know what to do in case of an emergency.” (Interview 3)
	Support chronic disease management • Identify students with chronic disease • Educate how to properly take medication and utilize medical devices • Share information related to condition • Provide resources for care (e.g., appointments, transportation) • Destigmatize health conditions	“I see them [community health workers] supporting individual referrals and those who present these initial concepts. Maybe the nurse identifies a chronic patient. This student always comes in complaining of their shortness of breath, ‘could you please follow up with them and their family to establish an asthma management plan?' I see them like that. (Interview 6) “[Community health workers can] Absolutely help them [students] with chronic health such as the program I worked with at [organization]. Like the pediatric asthma, updating them on information of testing, following up to make sure they get the right providers.” (Interview 13)
	Address social determinants of health • Conduct home visits to assess home environment • Social determinants of health screening • Link students and families to resources	“I think that [community health workers in schools conducting home visits] would be a good thing because it kind of reflects back to what we were doing. That educator can be doing home visits and then bringing that information back to the schools. For example, assessing the home environment, we would do environmental checklists. We would go through the home and rechecking for carpeting - all these things that would trigger people's asthmas. I think for the school, I'm thinking that it would also be beneficial for the educator to sort of have these tools, these assessments screeners to gauge where each family is as afar as their environmental needs, socioeconomic situation.” (Interview 7) “I worked in a school several years ago, so it's a lot of things that a community health worker could possibly help. You have children that come to school [and] they're hungry. You don't understand why they're hungry. To see the school system really grow from a community health worker being there, “Johnny is not hungry today and wonder why is that?” “Well, the community health worker saw that the amount of food stamps that the mother was getting wasn't sufficient for the home, so we recommend them to a food pantry.” A community health worker is valuable in a lot of different ways because we could probably get something out of the parents that the teacher or the principal can't get out of the situation.” (Interview 12)
	Build trusting relationships with students • Engage with students at school events (e.g., assemblies) • Maintain confidentiality • Show students that they are there to support them • Share knowledge	“I don't know if they still do that, they would have assemblies [and] the seventh and eighth grade [students] would attend. Maybe it's like once a month or every couple of months, a community health worker would just open up the assembly to certain grades and talk about why they're there, what they represent, what they could possibly bring to the table if you're going through this. This is not going to be shared with a teacher, a student, or your parents to make them feel comfortable.” (Interview 14) “It [community health worker-student relationship] has to happen organically. I don't know what specific avenue that would be because kids are very great judges of character. You have to be genuine.” (Interview 11)
	Support school staff • Be present in classrooms for observation • Support school nurse when not available in the school • Address students' medication needs • Back up for teacher assistant and student aides • Provide training to staff	“Having them [community health workers] in their classrooms honestly. I recall you would have the principal sit in the class to observe the school or the teaching. Sort of like that, I envision that educator doing rounds like we do in the hospitals. Having that kind of thing where the educator has some sort of schedule where in one day that educator will spend a few moments in each classroom. It doesn't even have to be an actual role but just sitting and observing.” (Interview 7) “I know it's across a lot of the school systems a nurse can't be there, but a community health worker can fulfill some of those roles in that absence and there are more of us than it is of them, so why not utilize them?” (Interview 3)
Collaborations to support work	Collaboration with school nurse • Nurse as clinician and CHW supporting outreach and data collection of student health information • Nurse to champion CHW and program • Work together to share health information with school • Nurse identifies health issue and CHW provides resources	“The nurse should be a partner [to the community health worker] where the nurse will be more on the clinician side, but then the community health worker can do more the reaching out, data collections for the nurse, having surveys, identifying which kids have certain chronic situations, sort of more like a support that can link clients to them too.” (Interview 7) “When a problem is identified, it's not just directed to the counselor or to the nurse, but just drawing in the community health worker because everybody's role is different. The community health worker may be able to bring other resources or add some that they didn't know about or was not familiar with. I just think working hand in hand and doing it collectively on a student that's been identified as having a particular problem.” (Interview 11)
	Collaboration with teachers • CHW train teachers on how to support students with chronic disease • CHW check in with teachers about students' progress • Both CHW and teachers identify school needs • Both CHW and teachers coordinate health education for students together	“That chronically ill student's primary teacher, they [community health workers] need to be working closely with. ‘We have identified you have three chronically ill kids in your classroom. So what do you need from me or how do you need me to educate you on how to treat this chronically ill student?'… You have someone on hand that you can say I need you to come up. I think he may be having an episode. And you come up and check them. And she can continue to do her job. And you're doing yours.” (Interview 2) “With the teachers who are in these classrooms, they know these students better than anybody. You want to be able to give people respect in the roles that they carry, but also know that we're in this together. We are a partnership. If we're trying to get to shore and you have an oar and I have an oar, we've got to do this together or we won't go anywhere. So let that nurse be that leader, that person that they connect with. Of course, administration needs to know that they're there but then those teachers too and be able to check in with the teachers. ‘Do you think does the class need an education session? Does this need to happen?' Not pulling the children out, make it a part of the everyday culture so even when they see somebody having an asthma exacerbation, they know what to do in case of an emergency.” (Interview 3)
	Collaboration with other staff • Administration/Principal • Behavioral team • Social worker	“Overall, it would be the nurses, principal because that should be a good backup, behavioral health team or counselors, teachers, as well as parents. Who else? I think all staff, honestly, they have to make sure to build that rapport with everybody to be known and for the staff to know something's happening or there's an emergency, or we have a question in regard to a student. They could also be that part of like contact in case somebody else is busy.” (Interview 5) “It has to be someone that can say, ‘let's have a conversation. Let's see what's going on in his school. What are some of the deterrents in this school that needs to be addressed and then have them address it?' I really believe that the CHW has to work closely with the principal and the parent advisory board.” (Interview 2)
	Collaborations with parents/families • To set goals for student/community health worker interactions • To prepare for student to receive care at school	“I feel like the parents have the best interest of the child at heart. So getting parents involved teachers, and like, administration to identify, what are we trying to do to make our students better? Like, what else can they learn? What else are they able to retain? And what else are we able to provide them so that the success rate for our students is where we want it to be and where they're able to obtain.” (Interview 4) “When they [a student] are chronically ill, the parent is overwhelmed. ‘Now you have me. Any questions you may have call me, and I'll help out. If you're going to bring the kids, the 504 plan allows any child to come into the school and carry their own medicine and administer that medicine to themselves.' A lot of people are hesitant to letting a four-year-old or a five-year-old administer their own medicine, which they probably can. I encourage the parents have yours in a Ziploc bag. Put it in his book bag. He has a right to carry it on him. If he doesn't know how to use it, then someone can help him use it.” (Interview 2)
	External collaborations • Health system • Community health groups • Physicians	“[Important for community health worker in school] To always aim to do professional development, attend trainings, join community groups – community health educated groups. There is a lot of them nationally but also locally, state-wide. Learn about what's going on in your profession, and there is no stupid question. I always say this - always ask questions.” (Interview 7) “In school, the nurse needs to be our partner, but then also who do they [community health worker] link back to like outside of the school system, is it the hospital that they're working for? Is it a certain clinic? Have a point person there so you don't have to go through a lot of bureaucracy. ‘This is the CHW supervisor of the CHW who's working in school A. You can reach back to them for protocols and processes, but we all need to be on the same page of what this is going to look like.'… There needs to be a person that supervises the CHW who understands the dynamic of maybe having to go through the home with the child to do a little education at the home if it's allowed and things of that nature. So and that would still fall under the hospital system or the clinic that they're attached to, to be that support.” (Interview 3)
Key steps for integration	Establish specific responsibilities	“The school should start really thinking on what would be this specific roles that they're really in need of that maybe a CHW can also be a part of - not so much takeover because there's certain training that needs to be done for a lot of things.” (Interview 5) “There definitely has to be a plan in place and a goal that can be achieved by both the school and the community health worker [for the community health worker to be effectively integrated into a school]. Maybe the school has a handful of students that have behavioral issues or there are students identified in the group that may have social issues Depending on what they're trying to achieve in, or if it's for all students, so just identifying the common goal.” (Interview 4)
	Introduce community health worker • CHW to introduce themselves and their role to staff, students, parents • School staff (e.g., nurse) to introduce CHW to teams and families	“When you have these orientation days or open house days in schools, having that presence of the Community Health Worker is key. On report card pick up days, having a table set up where you talk about your role as community health worker. “Why am I here? I'm here to connect you to this or that.” I'm even thinking at that point that educator can sort of survey people and ask the parents what they want to do.” (Interview 7) “Before a community health worker shows up, they [school staff] could start telling their team in those meetings, like “There's this program coming up. There's a community health worker that might be a part of our team.” Start doing that introduction [to the team] as well even before the CHW shows up. Just so they could be aware.” (Interview 5)
	Develop familiarity with school environment • School culture • School schedule/events • Classrooms, principal's office, and nurse's office	“[School should share with community health worker] what type of students they have. Some schools deal with more students that deal with homelessness, trauma, or low-income communities. If they're a dual-language school. As well as where things can be found, or what building they would be located. Because some schools have more than one building. Give them the main thing they need to know, like where's the principal's office? Where's the nurse's office? Where's the counselor's office?” (Interview 5) “You want them [community health workers] to be familiar with the school, the day-to-day of the school because that's how you create a sense of community. You just walk in not knowing the culture, you need to be familiar with the culture of the school.” (Interview 3)
	Develop familiarity with school population • School demographics • Academic performance • Common challenges faced by students	“I think it [the school] should give information like some of the negativity that the kids are going through, the number of each class, what they have for lunch, what are they doing as far as recess, their test scores because that plays a big part. If you're going to help our school, you need to know what's happening in the school. If you don't know how many how many eighth graders that's been graduating out of a class of 300 every year, why don't you know that? Why don't you know how many are getting A's? Are almost all your students just getting by? Are you just passing them on with D's because you're just like you're tired? I don't know if they would want to give us the information, but I would love to get the information because we wouldn't know how to help. What areas would we know how to help with if we don't have the information?” (Interview 10) “[Community health worker should know] Age range, which I'm sure the school would tell you that based on like elementary or middle. Definitely the demographics because you want to know how do I approach these people? How do these people feel in terms of like a community? For example, I know that a lot of African American communities aren't as trusting of like hospitals and healthcare systems. If I know that I'm coming from that system, then it's a certain way you have to approach. I definitely think age, demographic are the two big ones.” (Interview 11)
	Establish support system	“The work environment can help or can be an obstacle for the community health worker to develop these skills. I think having a good working environment where the community health worker feels comfortable and welcome - that's very important. Not like someone outside the school - that is part of the school. Making sure that it's really part of the team that they have the share of the school. That will be very helpful.” (Interview 8) “I feel like one of the only ways around that is to have a champion [within the school]. It has to be at least one person who actually believes in what you're doing or who is familiar with what you're doing that also has enough pull to make things happen. Sounds like an extremely difficult thing to do, but you have to have at least one person that's willing to be, ‘let's give this a shot.' That's what we have. It was really hard to get in until we found one person.” (Interview 11)
Characteristics of successful community health workers in schools	Familiarity with the community surrounding the school	“Successful [community health worker] is knowing the community they're working. They need to know what they have around what service they have to offer the service to the community. They need to be knowledge of the community they are serving.” (Interview 9) “If you're going to go along the definition of community health worker, you'll be able to find your person… You can create high school diploma or an equivalent, and at least so many years' experience doing community work or in another setting… We just know that they have some kind of community connection prior to jumping into this role because you have to be knowledgeable with the community that you're serving.” (Interview 3)
	Have relevant experiences for role • Working in community health field • Working with children and people	“I think if you have prior experience working with children [can prepare to support a school] because it's not easy. It's not easy dealing with children, especially if there is an issue that's going on. We need to have experience working with children, and not just your family because you deal with your family in a different way than you would deal with the children at school.” (Interview 12) “As long as they [community health worker in school] have a year of experience in the field, I think that would be helpful. Whether if it's doing home visits, you're working inside a hospital and you work with the clients, or you have that experience in outreach. Just a year experience of just being a CHW I would think would be a lot more helpful than having new ones that don't know what to expect or end up taking the role a little differently. Because there's that possibility, they might think, ‘oh, well, I'm going to be like a teacher's assistant.' You know, sort of like, knowing exactly what a CHW is, and what their role is would be helpful.” (Interview 5)
	Have specific qualities essential for role • Enjoy working with children • Positive • Flexible • Honest • Respectful • Open to learning	“You will need to like kids to be at that school. There're already enough people that probably work there that either are burnt out and they're just don't have the patience for kids as well. So they need to be able to like kids.” (Interview 5) “[Community health worker in school needs to have] Compassion, respect, willing to learn. Even if my organization is giving me lessons and seminars on how to deal with a certain situation, the school might have a way that they see might work better than what I learned, so a willingness to continue to learn. You're never going to learn everything. There's always room to grow, so a willingness to learn.” (Interview 12)
	Have relevant skills for role • Leadership • Communication • Establish professional boundaries • Resourceful • Cultural competency • Computer literacy	“I think having a healthy understanding of technology - the basis of Microsoft, Word doc, Excel, and PowerPoint – being very imperative skills to have with you. If the organization can train you on how to use those platforms, I think that's an additional time and effort that doesn't necessarily need to be instituted into this process. It would be good for them to come with that skill set.” (Interview 6) “Again, I would go back to being open, being able to communicate. You know how you hear different supervisors say I have an open door policy, the health worker more or less having an open door policy, being able to communicate with all levels and different environments.” (Interview 14)
Training for community health workers in school	Training on community health worker core skills • Communication • Cultural humility • Adverse childhood experiences • Patient privacy/HIPAA • Motivational interviewing • Community outreach	“I didn't have any prior experience entering the CHW role, so I feel like proper training that it would be possible for that CHW [in school] to still be effective as long as they are properly trained. And I guess them doing their homework to see what resources are available in that community and then again it goes back to being trained on how to look for resources and who to connect with.” (Interview 1) “More trainings like with CPR are important, like emotional first aid, some computers, [and] computer skills for sure. We have a special training for community health workers, which basically shows how to approach to patient: how to be respectful to clients, the things we can say, the things we are not allowed to say, the things we might face. That kind of training I think it's mandatory.” (Interview 8)
	Training on health/wellbeing and medical topics • Early childhood development • Chronic diseases • Nutrition • Mental health • Cardiopulmonary resuscitation (CPR) • First aid	“If I knew you were going to ask me this question [on certain trainings CHWs should complete before supporting a school community], I would have every training that I have had. Early childhood development, ACEs [adverse childhood experiences]… Different abuse has a lot to do with how a child learns. Mental health - one year, I took two mental health classes and I'm like, ‘wow.' It's a lot of trainings that we really need to focus on for the children's health - mental health, physical health, all of that plays into it.” (Interview 12) “If they're [community health workers in school are] trained in asthma, they can help the kid. They can teach the student on how to properly take their medication, how to properly use their inhaler. Because that's one thing that we did notice coming from teaching kids with asthma is that the majority don't know how to properly use their inhaler.” (Interview 5)
Assessment of progress or impact	Evaluation tools • Survey of students, families, and staff • Reports	“I guess you'd have the parents to have a survey before and after as well as the teachers. The parents and the teachers would have a survey after. After you completed your work or ongoing. It could be actually ongoing… If you want to have a survey to see if we're doing what we say we're going to do, then you would definitely need a survey for that.” (Interview 13) “Some kind of report back would be good. I mean we would enter information to the database or even give a report about some experience - what happened this week or what is your plan for this week. At the end of the week what happened with that? Almost like the goals we set for the week and at the end of the goal we go through those check-ins with the CHW to see where they are and that list of things.” (Interview 3)
	Data to collect or utilize for evaluation • Interactions with students Work progress Observed changes among students interacting with CHW	“If you're dealing with a student whatever issues are going on, it's something that you're going to log… Keeping everything that is going on with a particular student logged in from beginning to end - when the student came in, what they talked about, what you did, what was your suggestions, how did they feel, what did they do. Just everything so that there is a track record so that you do have something to refer back to so that you know if progress is being made or if you're going backwards or if you need to do something different… Write it all up.” (Interview 14) “Definitely [can get feedback from community health worker] through the data collection also via meetings, quarterly meetings, bi-weekly meetings. One of the things that we started doing with our research assistant to evaluate some of the stuff is that when I have my check-ins with my educators on a bi-weekly basis, we talk about caseloads, audits. The research assistant joins once a month and collects anecdotal information from the educators - challenges, successes with the clients that we have. He jots down those notes, and he incorporates those findings on our reports for funders but also for the Board to inform not just numbers, but also having a story to the families that we're serving, the clients that we're serving.” (Interview 7)
	Signs of successful programming • Improvements in academic performance • Decreases in absenteeism • Positive changes in behavior • Decreases in medical emergencies • Integration of CHW in classrooms • Referrals to CHW	“Just them [community health workers] being in the classroom should already be a success. Like, you're like, ‘yay, they're allowed to be in the classroom.' Even if it's just for an hour or two, like that's already a success.” (Interview 5) “If it was a child that was always absent and now, they are coming to school on time or regularly because they were sick or whatever the case may be, I think that if the child was present in school and they're managing their disease or whatever, I think that shows that the intervention is working.” (Interview 1)
Potential challenges	Challenges from within school community • Pushback from school community	“I think the roles [of community health worker] have to be clearly identified. A lot of times, clinicians tend to be a little bit territorial with the community health workers. We do have a lot of champions out there that believe the community health worker model is effective. It's a team effort. It should be a team effort making sure that the rules are clearly aligned and reflected on whatever process you're going to have.” (Interview 7) “[Community health workers can be integrated successfully into schools] With the support of the teachers and the staff because sometimes it happens to me that they feel threatened that you're going to take their responsibilities, but it's completely different. It's because we're not social workers. We're not nurses. We're not teachers. We have a wide knowledge about resources. Sometimes if the staff do not work diligently, that might affect the performance of the community health worker on schools.” (Interview 8)
	Challenges related to scope of work • Overwhelming student cases • Maintaining confidentiality of student information • Existing restrictions within school system • Sustainability of work • Communication with people of various backgrounds	“I think the only other issue that might arise is parents possibly wondering who this person [community health worker] is, what they're doing there. You want to know who's being hired to work in a school with children. You also want to make sure that they know how to properly address and interact with children because some people are great at their jobs, but everyone doesn't work well with kids. I think that's the big challenge is make sure whoever it is, is prepared to work with kids or teenagers cause that's a thing too.” (Interview 11) “I think it is challenging to work in the school system. We tried it when we were doing our asthma interventions. There's a lot of bureaucracy out there, but I think it's doable. I think if you have the right team and the right people on the table, you should be able to work something out and have a great partnership.” (Interview 7)

### 3.1. Roles and responsibilities of CHWs in schools

Participants identified various responsibilities that school-based CHWs can perform to strengthen health promotion in schools, including educating school communities about health, addressing social determinants of health affecting students and families, supporting chronic disease management among students, supporting school staff, and building trusting relationships.

Participants emphasized CHWs' main role is to support the school community. They described a key responsibility to educate students, families, and staff about health, including chronic health conditions, general health (e.g., nutrition, vaccinations), and school health policies. Participants suggested that CHWs can also address social determinants of health among students and families through screenings, home visits, and linkages to health and social resources. One participant shared: “*Johnny [a student] is not hungry today and wonder why? The CHW saw that the amount of food stamps the mother was getting wasn't sufficient for the home, so recommended them to a food pantry.”* Additionally, participants described how CHWs can serve as resources for students with chronic health conditions through education, linkage to resources and care, and advocacy for students to properly manage their condition(s) at school with section 504 plans. For example, participants stated that CHWs can teach all students about specific health conditions. One participant explained, “*You do it as a teachable moment. You talk to the class so everybody is aware because not only the person who has asthma should know about how to control it, but those around him should know what to do in an emergency.”*

Along with supporting students and families, participants shared CHWs can support school staff including nurses and teachers. For school nurses, CHWs can assist with responsibilities that do not require licensing (e.g., administrative work). One participant said, “*The educator [CHW] and the nurse should be partner[s]. The nurse will be more on the clinician side, then the CHW can do the reaching out, data collections for the nurse, identifying which kids have certain chronic situations.”* Participants highlighted that supporting school nurses can involve fostering communication, tracking student health data, and reviewing medication management plans with students. Additionally, they suggested CHWs can support teachers by visiting classrooms and monitoring students for health emergencies. For students with chronic health conditions, CHWs could educate teachers about supporting these students and responding to health emergencies.

For CHWs to support the school community, participants indicated it is important for CHWs to build trusting relationships, especially with students. Methods cited for organically developing relationships with students and staff include having a consistent presence, maintaining confidentiality, and sharing knowledge. One participant suggested, “*Maybe it's once a month or every couple of months, a CHW would open up the assembly to certain grades and talk about why they're there, what they represent, what they could possibly bring to the table.”*

### 3.2. Collaborations to support work

Collaborations with school staff, parents/guardians, and external organizations were identified by participants as critical for enabling CHWs to successfully encourage healthy environments within schools.

Participants recommended CHWs work closely with school staff to address student needs and build a culture of health in schools. They described a partnership with the school nurse in which both collaborate to identify and address health issues among students, such as through communication between students, parents, and school nurses facilitated by CHWs; care provided by the school nurse; and resources identified by the CHW. Additionally, participants indicated school nurses can play a critical role in CHW integration by introducing the role to the school community. One participant explained, “*A CHW needs a vehicle to get into the space. The most logical part is the nurse. The nurse introduced them and said, ‘This is the CHW. When I'm not here, this is your go-to person.”'*

Along with the school nurse, participants suggested a collaboration between teachers and CHWs, since “*teachers who are in these classrooms know students better than anybody,”* as one participant said. Such collaboration could entail coordinating health education for all students and supporting students with chronic conditions. Participants also emphasized the importance of CHWs working with all school staff (e.g., administration, behavioral health team) to better understand their role and how to maximize their work in addressing school needs. The CHW can train all staff to identify students' health needs and respond to medical symptoms or emergencies. One participant stated, “*[CHWs] have to make sure to build that rapport with everybody to be known and for the staff to know something's happening or there's an emergency, or we have a question in regard to a student*.”

Participants also highlighted an essential collaboration with parents/guardians of students with chronic health conditions. Participants envisioned CHWs learning from parents about children's needs and setting goals together. Additionally, they suggested CHWs can work with parents to understand policies that support their children's right to manage their condition at school. One participant shared, “*For a healthy environment, the parent and school know from the 504 plan and IEP things this kid is allowed to do. One of my biggest issues was that teachers did not want to take out time of a lesson to give a treatment. [CHW] will help support the laws and the benchmarks that [school district] has.”*

Outside of the school community, participants proposed CHWs develop external collaborations with health systems and community organizations. These collaborations would serve dual purposes – to expand resources to the school community and to support CHWs' ongoing professional development.

### 3.3. Key steps for integration

Participants identified specific steps that would be needed to successful integrate CHWs in schools: establish responsibilities of CHW, familiarize CHW with the school environment and population, introduce CHW to the school community, and establish a support system for CHW.

Participants expressed that expectations of CHWs should be well-established before the program's start. These expectations may depend on students' needs, as one participant shared, “*Maybe the school has a handful of students that have behavioral issues or students identified in the group that may have social issues. Depending on what they're trying to achieve in [working with CHW], or if it's for all students, so just identifying the common goal.”* To identify CHWs' responsibilities, participants stated it is important for the CHW to understand the school community and its needs. They advised CHWs should familiarize themselves with the school, including existing health programs, policies, and culture, and the student population, including demographics, academic performance, and needs. One participant stated, “*[The school] should give information like the negativity the kids are going through, the number of each class, what they have for lunch, what are they doing as far as recess, their test scores. If you're going to help our school, you need to know what's happening in the school.”* Some participants suggested CHWs can perform a needs assessment to identify needs and risk factors of students, parents, and school staff, which can inform CHWs' responsibilities and priorities.

Participants indicated it is also important for CHWs to be introduced to school staff, students, and parents during integration. Participants suggested CHWs join staff meetings and parent-teacher conferences to build relationships within the community, as such introductions help establish trust and awareness of the CHW role as well as enable CHWs to gain more familiarity with the school community. One participant elaborated, “*When you have these orientation days or open house days in schools, having that presence of the CHW is key. That educator can survey people and ask the parents what they want to do.”* Participants also recommended school leaders and nurses take initiative to introduce CHWs to their teams to integrate CHWs into the school setting more seamlessly. Participants recognized that it is important for CHWs to be part of teams and acknowledged within schools for their work to be effective. “*You have to have a champion,”* said one participant. “*It has to be at least one person who believes in what you're doing or who is familiar with what you're doing that also has enough pull to make things happen.”*

### 3.4. Characteristics of successful CHWS

Participants identified four key characteristics that CHWs should possess to positively impact school communities: (1) familiarity with the community surrounding the school, (2) related work experience, (3) essential professional skills, and 4. specific personal qualities.

Participants stressed CHWs should be knowledgeable about the community surrounding the school, including demographics and nearby resources. “*[Success] is knowing the community they're working,”* said one participant. “*They need to know what they have around, what service they have to offer the service to the community.”* Having a connection to the community prior to beginning the CHW position, such as being a member of the neighborhood, was reported as useful. Also, participants indicated it was important for CHWs to have prior work experience in community health and/or with people. Previous experience and confidence working with children is valuable, particularly as CHWs support students during formative years of social, physical, and cognitive development. One participant stated, “*What you do with a student can be lifelong*. *When you get the experience of dealing with different temperaments, different cultures, different things that students have gone through, it just prepares you better.”*

In addition, participants highlighted several professional skills and personal qualities necessary for serving in schools. Relevant skills included leadership, communication, resourcefulness, problem-solving, and reliability. Participants reported CHWs should also be culturally competent, specifically approachable, non-judgmental, and able to communicate effectively with others from different backgrounds. Computer literacy was also identified as valuable, with proficiency in programs like spreadsheets and presentations useful for tracking progress. Because community health work can be emotionally challenging, participants emphasized the importance of self-care and the ability to maintain professional boundaries. Additional personal characteristics mentioned were positivity, flexibility, honesty, respect, compassion, and willingness to learn. Participants also emphasized CHWs should enjoying working with children. One participant said, “*You will need to like kids to be at that school. There [are] already enough people that work there that either are burnt out and they're just don't have the patience for kids.”*

### 3.5. Training for CHWs in schools

Participants discussed the importance of training to equip CHWs with the knowledge and skills necessary to provide CHW services in schools, focusing on two primary areas: core skills and health/medical topics. They suggested training can be delivered through different methods, including online modules and shadowing.

Participants identified core skills training should include communication, de-escalation, cultural humility, and patient privacy. Additional skills cited as important were motivational interviewing, community outreach, resource identification, and evaluation. Depending on students' ages, participants recommended that CHWs be prepared to work with children, including training in early childhood development and adverse childhood experiences. One participant described, “*[Make] sure that the CHW is trained to be in the environment that the facility wants them to be in. So if there's early childhood, learn how to deal with a child.”*

Participants also voiced that training on varied health-related topics should be required to enable CHWs to focus on relevant aspects of student health. They stated CHWs should be trained in general health topics (e.g., nutrition, mental health) and chronic diseases prevalent at the school (e.g., asthma, diabetes), so they are prepared to help students with those conditions manage their care. One participant summarized, “*We're talking about students, so anything that has to do with behavioral issues, how health issues affect children in school, how does it affect their learning depending on what the health issue is, how you approach children who are dealing with certain health issues, anything that opens the door for better understanding, better communication, better ways to be able to deal with students in an open environment.”* Furthermore, participants advised school-based CHWs should know first aid and cardiopulmonary resuscitation in case of emergencies.

### 3.6. Assessment of CHW impact

Participants shared their recommendations for assessing the impact of CHWs on school communities, including evaluation tools, data collection, and indicators of success.

Participants proposed conducting surveys to obtain feedback from the school community on the quality of CHW services. They identified the utilization of CHWs as an additional indicator of success, such as in-house referrals to CHWs and the inclusion of CHWs in classrooms. Beyond survey and utilization data, participants suggested monitoring academic and health indicators of successful programming. For example, they described CHWs' positive impact may be signaled by changes in student outcomes such as improved academic performance, decreased absenteeism, favorable behavioral changes, and fewer medical emergencies.

Participants also recommended CHWs collect both quantitative and qualitative data when recording progress. For example, CHWs can document general feedback and details of their student interactions, as one participant explained: “*Keep everything that is going on with a particular student logged in from beginning to end – when the student came in, what they talked about, what you did, what was your suggestions, how did they feel, what did they do*. *So you have something to refer back to so that you know if progress is being made or if you need to do something different.”* Participants suggested online databases can be used to track weekly progress and inform follow-up with students as necessary. To summarize the work and impact of CHWs, participants communicated that CHWs can compile formal reports to share with school administrators and program evaluators using data from surveys, documentation, and school outcomes.

### 3.7. Potential challenges

Participants anticipated school-based CHWs may need to overcome challenges in their role arising from within the school community or related to their scope of work that could hinder their integration.

Participants identified a major challenge to successfully integrating CHWs into schools may be pushback from the school community. Specifically, existing school staff may perceive CHWs as threats to their own positions, and parents may be wary of having an unknown person work with their children. To address this challenge, participants suggested that CHWs be prepared to explain their role and communicate their qualifications. One participant explained: “*Just be open. Be transparent and be willing to answer questions. Just say, ‘I'm here to partner with you in this process because we have the same common goal to make sure the student is taken care of and is healthy in mind, body and spirit.”'*

Participants also shared that CHWs may face challenges related to their scope of work, such as communicating with people of various backgrounds and maintaining student confidentiality while being a mandated reporter. Other potential challenges related to CHWs' scope of work included overwhelming numbers of student cases, emotional toll of difficult situations, and existing limitations within school systems (e.g., funding, resources). Participants stressed CHWs should rely on their support system (e.g., colleagues, supervisors) and other resources (e.g., mental wellbeing trainings) to overcome these challenges.

## 4. Discussion

This study is the first to describe the perspectives of experienced CHWs about the potential roles and impact of CHWs in schools. Much of the existing literature focuses on CHWs in clinical or community settings, rather than in schools. This study explores important considerations for integrating CHWs in schools to support student health and healthy school environments.

The proposed services for CHWs in schools in this study are consistent with literature exploring CHW roles in other settings. Studies describing CHWs in primary care settings found they commonly conducted health assessments, made connections to community resources, and provided health education and coaching (20, 36, 37). Literature on CHWs in community settings reported similar responsibilities as in primary care, including advocacy for clients' health and linkage to health care services (38, 39). Participants in this study detailed additional responsibilities specific to school settings, such as education on school health policies and guidance to all school community members–not solely individuals with health conditions–about responding to health emergencies. While few studies have detailed the responsibilities of CHWs in school-based programs, a systematic literature review of CHWs in schools observed the majority of interventions reported positive outcomes, with nearly all successful interventions involving CHWs providing health education or coaching to students (13). Other research has identified needs within schools that CHWs can address; for example, one study showed school staff and parents viewed each other as responsible for providing nutrition education to students (40). CHWs can fill this role and empower both school personnel and parents to support healthy behaviors among students (41).

Successful programming in schools is a multifaceted process that includes developing collaborations. Participants in our study highlighted the importance of CHWs partnering with school nurses. Literature has identified CHWs and school nurses share overlapping goals, including providing care coordination and bridging gaps between healthcare and education (42). By leveraging their unique trainings and skills, CHWs and school nurses can complement each other to deliver health education as well as perform health screenings and contact tracing to prevent infectious disease spread (42). Both this study and prior research recommend that CHWs working with children also collaborate with parents to address social determinants of health (38). Whereas participants in this study stressed the importance of external collaborations to expand resources and support professional development, existing literature further highlights how relationships with local organizations can enable CHWs to reach more individuals and increase awareness of CHW services within communities (43).

To effectively integrate CHWs into schools, prior literature has emphasized the importance of having a champion for the CHW program, aligning with participants' recommendation to establish support systems (44). This study primarily focuses on actions that CHWs can take for successful integration, while previous literature concentrates on how other individuals can prepare to work effectively with CHWs (44). Specifically, it is important to inform clients about CHWs to increase their readiness to utilize these services. In school settings, this is akin to participants' suggestion that school leaders disseminate information about the CHW to the school community. Such introductions are important to prepare school members to optimally utilize the CHW, as CHWs have been shown to have greater readiness than clients and higher odds of anticipating themselves capable of performing services than clients reported a need for such services (45, 46).

Most recommended skills and qualities proposed by participants for CHWs working in schools align with those reported in existing literature. Specifically, studies describe it is important for CHWs to be attentive listeners, understand community needs, and function without biases to perform their duties effectively (36, 47). A major strength of CHWs, as described in this study and other research, is their ability to relate personally to clients. Participants recommended that school CHWs be members of the surrounding community to understand the school's needs, similar to research describing that a key asset for CHWs to build trust is sharing life experiences with clients from the same community (47, 48). While not highlighted in this study, recommendations from other research to consider for implementing CHWs in schools is to provide enabling work environments for CHWs with a manageable number of tasks and easy access to supplies (49, 50).

The trainings in core skills and health-related topics recommended in this study, including communication, cultural competency, and mental health, are similar to those described in prior research. Literature supports that evidence-based trainings, including cultural sensitivity, are important for CHWs to work effectively (51). Other specialized trainings from prior studies to consider include program management, advocacy, and evaluation (43).

In terms of evaluating the impact of CHW programming in schools, participants in this study recommended recording CHWs' interactions with the school community, similar to previous research highlighting the importance of tracking the number and quality of interactions between CHWs and clients, team members, and external organizations (52). While not emphasized in this study, other relevant metrics of success described in the literature include cost-efficiency, sustainability, job satisfaction, and CHW involvement in other areas such as school policy (36, 44, 53). Fewer studies have endorsed specific evaluation methods; however, some recommend mixed-methods approaches, aligning with participants who indicated qualitative data can capture nuances while quantitative data can provide summative assessments of effects as well as inform future programs and grant applications (52, 54).

Finally, this study adds to the literature about challenges CHWs may face in their work environment and responsibilities. Participants reported that inadequate resources and overwhelming workloads, which literature also identifies as major barriers for CHWs, may negatively impact work quality (51). Prior studies also observe excessive work demands can lead to stress, social isolation, and burnout, which aligns with the emotional toll that participants described school CHWs may experience (51, 55). Further, participants' comments about CHWs facing pushback from the school community expands upon the limited research about CHWs being potentially unwelcome in the workplace. CHWs working in health care teams have previously shared the importance of dedicating time to building trust with team members during integration (56). As team members may be unaware of how to partner with CHWs, preparing them to work alongside CHWs can help resolve any disruptions related to the integration of CHWs into the team (44, 56).

An important strength of this study is that it elicited rich data from one-on-one interviews with CHWs to thoroughly examine their reflections and ideas. This study highlights the perspectives of CHWs, who have unique insights from their personal work experiences into their role and how it may be applied in school settings. However, this study is not without its limitations. While theme saturation was reached, this study may have limited generalizability due to the sample size and location of participants, with all in the Chicagoland area and most affiliated with a health system. The results may not apply to non-urban or rural communities, although they are foundational to efforts to integrate CHWs in Chicago schools. Furthermore, participants' status as paid employees and demographics (e.g., race, ethnicity, socioeconomic class) are not reported, which may shape their perspectives on integration into schools. Selection bias and recall bias may have also influenced findings. Future studies can examine perspectives from a wider range of CHWs and other relevant stakeholders across communities and regions.

While schools may have an ethical obligation to provide health support to students (57), the schools that would likely derive the most benefit from CHWs may be the least likely to have the capacity or resources to integrate them, which could potentially exacerbate inequalities. Implementing CHWs into under-resourced schools would require obtaining funding, which can be achieved through budgeting administrative dollars toward CHW services, obtaining Department of Education funding, or reimbursing their services through state Medicaid plans if the state acknowledges CHWs as Medicaid providers (58). For schools that lack infrastructure, the implementation of CHWs in schools would require developing unique partnerships with health systems or CHW-focused organizations to facilitate the hiring and integration of school-based CHWs.

CHWs play a valuable role in the health care system, increasing access to care by connecting vulnerable communities to health and social services. While CHWs interventions have positive outcomes in clinical and community settings, the implementation of CHWs in schools has been underexplored. This study engaged experienced CHWs to obtain critical insights on how CHWs can be integrated and utilized in school settings. The results can serve as guidance for schools integrating CHWs and states expanding school-based health services, particularly in the setting of the free care rule reversal by Centers of Medicare and Medicaid Services (59). This study's findings are especially relevant to clinicians, researchers, and school leaders responsible for ensuring a healthy environment for students within schools.

## Data availability statement

The original contributions presented in the study are included in the article/Supplementary material, further inquiries can be directed to the corresponding author.

## Ethics statement

This study involved human participants and was reviewed and approved by the University of Chicago Institutional Review Board. Written informed consent for participation was not required for this study in accordance with the national legislation and the institutional requirements.

## Author contributions

JC, TD, KF, SI, and AV acquired funding for this study. JC, TD, KF, SI, MK, and AV conceptualized and designed this study. MK collected data. NY, MK, LG, and AV analyzed and interpreted data. MK and NY wrote the first draft of the manuscript. All authors critically revised the manuscript.
