# Supplementary material for: Community health workers' perspectives on integrating into school settings to support student health
Source: Front Public Health. 2023 Jun 21;11:1187855. doi: 10.3389/fpubh.2023.1187855 (PMC10320159; doi:10.3389/fpubh.2023.1187855)
Supplement: Supplementary file 1 [file Table_1.DOCX]

**Community Health Worker Perspectives on Integrating Community Health Workers in Schools**

**Interview Guide**

*We are developing a program to integrate a community health worker in schools to help build a culture of health within schools and address the health needs of students. I would like to hear your insight on how a CHW can be successfully integrated in schools to support care.*

1. *Overall role*

- What role(s) do you think a CHW could play in a school?
- What would success look like for a CHW serving a school community?
  - - How would you assess success?

1. *Focus on health in schools*

- How can a CHW support a culture of health / healthy environment in school?
  - - What actions should a CHW take to support a culture of health in schools?
    - Who should the CHW work with to support a culture of health in schools?
  - How can a CHW support students with chronic health conditions?
    - What actions should a CHW take to support students with chronic health conditions?
    - Who should the CHW work with to support students with chronic health conditions?
  - How can the CHW work with / partner with the school nurse to support a culture of health?

1. *Integration of CHW in school*
   - How can a CHW be most effectively integrated into a school?
     - What actions would it be important for the CHW to take?
     - What actions would it be important for others on the team to take? (like school admin, nurses, etc)
     - What actions would be important to undertake when the CHW is first starting?
   - What should a CHW prioritize in the beginning to successfully integrate into the school?
     - Over time, how should the priorities of the CHW change?
   - How would you know if a CHW has effectively integrated into a school?
     - What are early metrics of successful integration?
   - How should a CHW prepare for supporting a school community?
     - What training(s) should a CHW complete before supporting a school community?
     - What information should a CHW be given about the school and school community before they begin supporting a school?
     - What prior experience should a CHW have to prepare them to support a school?
2. *Relationship*
   - What would a successful school-CHW relationship look like?
   - What qualities should a CHW have to be successful in forming and nurturing their relationship with a school community?
   - What actions should a CHW take to develop the relationship with the school community?
   - What would a successful relationship between the CHW and school nurse look like?
3. *Monitoring*
   - How should the work of a CHW in the school be tracked?
   - How can the administrative project team and/or evaluation team get feedback from the CHW most effectively?
4. *Challenges*
   - What challenges may arise when integrating a CHW into a school?
     - How might these challenges be overcome?
     - How might a CHW prevent or overcome these challenges?
5. Insights
   - What should a CHW know before beginning their role in the school?
   - What advice would you give to someone beginning a new position as a CHW within a school?
